# Hypoxia releases S-nitrosocysteine from carotid body glomus cells—relevance to expression of the hypoxic ventilatory response

**DOI:** 10.3389/fphar.2023.1250154

**Published:** 2023-10-11

**Authors:** James M. Seckler, Paulina M. Getsy, Walter J. May, Benjamin Gaston, Santhosh M. Baby, Tristan H. J. Lewis, James N. Bates, Stephen J. Lewis

**Affiliations:** ^1^ Department of Biomedical Engineering, Case Western Reserve University, Cleveland, OH, United States; ^2^ Departments of Pediatrics, Case Western Reserve University, Cleveland, OH, United States; ^3^ Department of Pediatrics, University of Virginia, Charlottesville, Virginia, United States; ^4^ Herman B Wells Center for Pediatric Research, Indiana University School of Medicine, Indianapolis, IN, United States; ^5^ Galleon Pharmaceuticals, Inc, Horsham, PA, United States; ^6^ Department of Anesthesia, University of Iowa, Iowa City, IA, United States; ^7^ Departments of Pharmacology, Case Western Reserve University, Cleveland, OH, United States; ^8^ Functional Electrical Stimulation Center, Case Western Reserve University, Cleveland, OH, United States

**Keywords:** primary glomus cells, S-nitroso-L-cysteine, L-S-nitrosoglutathione, Smethyl-L-cysteine, S-ethyl-L-cysteine, minute ventilation

## Abstract

We have provided indirect pharmacological evidence that hypoxia may trigger release of the S-nitrosothiol, S-nitroso-L-cysteine (L-CSNO), from primary carotid body glomus cells (PGCs) of rats that then activates chemosensory afferents of the carotid sinus nerve to elicit the hypoxic ventilatory response (HVR). The objective of this study was to provide direct evidence, using our capacitive S-nitrosothiol sensor, that L-CSNO is stored and released from PGCs extracted from male Sprague Dawley rat carotid bodies, and thus further pharmacological evidence for the role of S-nitrosothiols in mediating the HVR. Key findings of this study were that 1) lysates of PGCs contained an S-nitrosothiol with physico-chemical properties similar to L-CSNO rather than S-nitroso-L-glutathione (L-GSNO), 2) exposure of PGCs to a hypoxic challenge caused a significant increase in S-nitrosothiol concentrations in the perfusate to levels approaching 100 fM via mechanisms that required extracellular Ca^2+^, 3) the dose-dependent increases in minute ventilation elicited by arterial injections of L-CSNO and L-GSNO were likely due to activation of small diameter unmyelinated C-fiber carotid body chemoafferents, 4) L-CSNO, but not L-GSNO, responses were markedly reduced in rats receiving continuous infusion (10 μmol/kg/min, IV) of both S-methyl-L-cysteine (L-SMC) and S-ethyl-L-cysteine (L-SEC), 5) ventilatory responses to hypoxic gas challenge (10% O_2_, 90% N_2_) were also due to the activation of small diameter unmyelinated C-fiber carotid body chemoafferents, and 6) the HVR was markedly diminished in rats receiving L-SMC plus L-SEC. This data provides evidence that rat PGCs synthesize an S-nitrosothiol with similar properties to L-CSNO that is released in an extracellular Ca^2+^-*dependent* manner by hypoxia.

## Introduction

Endogenous S-nitrosothiols, such as L-S-nitrosocysteine (L-CSNO) and L-S-nitrosoglutathione (L-GSNO), modulate the activities of numerous neural circuits in the central nervous system (Lei et al., 1992; Lipton et al., 1993; Lipton et al., 1994; Takahashi et al., 2007; Tegeder et al., 2011; Raju et al., 2015; Tarasenko, 2015; Nakamura and Lipton, 2016). Moreover, these S-nitrosothiols modulate numerous peripheral neural systems (Meller et al., 1990; Matsuda et al., 1995; Savidge, 2011; Lee et al., 2013; Tooker and Vigh, 2015; Gaston et al., 2020). The proposed mechanisms by which S-nitrosothiols exert their effects on neuronal systems include breakdown to nitric oxide and creation of iron-nitrosothiol complexes that activate soluble guanylate cyclase and down-stream cGMP-dependent protein kinase signaling events (Mellion et al., 1983; Travis et al., 1996; Severina et al., 2003; Lima et al., 2010; Martínez-Ruiz et al., 2013; Marozkina and Gaston, 2015; Vanin, 2019). In addition, it is clear that S-nitrosothiols exert their effects by transferring NO^+^ to sulfur atoms (S-nitrosylation) in a large number of functional proteins (Jaffrey et al., 2001; Joksovic et al., 2007; Foster et al., 2009; Lima et al., 2010; Rudkouskaya et al., 2010; Marozkina and Gaston, 2012; 2020; Anand et al., 2014; Pires da Silva et al., 2016; Wynia-Smith and Smith, 2017; Stomberski et al., 2019).

L-CSNO is an endothelium-derived S-nitrosothiol (Myers et al., 1990; Bates et al., 1991; Kukreja, et al., 1993) that is stored in cytoplasmic vesicles (Seckler et al., 2020). L-CSNO may be subject to exocytotic release within peripheral vascular beds (Davisson et al., 1996a; Batenburg et al., 2004a; b, 2009; Hashmi-Hill et al., 2007), and from lumbar sympathetic vasodilator nerves within the hindlimb vascular beds (Davisson et al., 1994; 1996a; b,c; 1997a; Possas and Lewis, 1997; Possas et al., 2006). Many of the pharmacological effects of SNOs, such as L-CSNO and S-nitroso-β,β-dimethyl-L-cysteine, are heavily reliant on their stereoisomeric configuration (Lewis et al., 1996; 2005a; b, 2006; Davisson et al., 1996d; 1997b; Travis et al., 1996; 1997; 2000; Ohta et al., 1997; Hoque et al., 1999; 2000; Lipton et al., 2001; Gaston et al., 2020). For example, the microinjection of L-CSNO into the nucleus tractus solitarius (nTS) decreases mean arterial blood pressure in anesthetized rats (Ohta et al., 1997), and microinjection of L-CSNO into the lateral (Davisson et al., 1997b) or fourth (Lewis et al., 1996) ventricles of freely-moving rats produce a complex array of hemodynamic responses, whereas similar microinjections of the D-isomer (D-CSNO) elicit minor responses. Although the identities of the functional proteins that may represent the stereoselective L-CSNO binding sites have not been fully characterized, we demonstrated that L-CSNO, but not D-CSNO, directly binds to and alters the activities of voltage-gated K^+^-channels (Kv-channels) that does not require S-nitrosylation of the ion channel (Gaston et al., 2020).

With respect to the control of breathing, it is known that microinjections of L-CSNO into the nTS increase minute ventilation in awake freely-moving rats by stereoselective-dependent mechanisms unrelated to the potential decomposition of L-CSNO to nitric oxide (Lipton et al., 2001). At the peripheral level, the carotid body contains, numerous excitatory and inhibitory neurotransmitters released by primary glomus cells (PGCs) in response to changes in ventilation (Prabhakar, 1994; Iturriaga and Alcayaga, 2004; Rey and Iturriaga, 2004; Bairam and Carroll, 2005; Kumar, 2007; Shirahata et al., 2007; Iturriaga et al., 2009; Nurse and Piskuric, 2013; Nurse, 2014; Gonkowski, 2020; Bardsley et al., 2021; Gold et al., 2022). However, there is no current agreement as to the identity of the primary neurotransmitter released by PGCs to drive the hypoxic ventilatory response (Eyzaguirre and Fidone, 1980; Iturriaga and Alcayaga, 2004; Nurse, 2005; Lahiri et al., 2006; Iturriaga et al., 2009; Gonzalez et al., 2010; Prieto-Lloret and Aaronson, 2017; Aldossary et al., 2020; Gold et al., 2022).

We have reported that arterial injections of L-CSNO (catheter tip positioned close to the carotid body artery) elicit dose-dependent increases in frequency of breathing (Freq), tidal volume (TV) and minute ventilation (MV) in freely-moving rats that are not mimicked by similar injections of D-CSNO (Gaston et al., 2020). We also reported that these L-CSNO-induced responses were reduced in rats with 1) prior bilateral transection of the carotid sinus nerves (CSNX) and 2) those receiving continuous intravenous infusion of S-methyl-L-cysteine (L-SMC), which reduces the ventilatory responses elicited by L-CSNO, but not those elicited by other S-nitrosothiols, such as L-GSNO (Gaston et al., 2020). Previous research has shown that nitric oxide has an inhibitory role in carotid body chemotransduction processes (Prabhakar et al., 1993; Chugh et al., 1994; Prabhakar, 1994; Trzebski, et al., 1995; Wang et al., 1995a; b; Summers et al., 1999; Iturriaga, 2001; Silva and Lewis, 2002; Rey and Iturriaga, 2004; Campanucci et al., 2012; Moya et al., 2012; Prabhakar and Peers, 2014). Consistent with this evidence, we found that arterial injections of the nitric oxide donor, MAHMA NONOate, inhibited breathing via actions in the carotid body (Gaston et al., 2020). This and our findings that inhibition of soluble guanylate cyclase reversed the effects of MAHMA NONOate, but minimally affected the excitatory actions of L-CSNO (Gaston et al., 2020), suggests that the excitatory actions of L-CSNO in the carotid body are not dependent on its decomposition to and activity of nitric oxide.

Our data led us to hypothesize that hypoxic challenges induce the release of preformed pools (perhaps vesicular stores subject to exocytosis) of L-CSNO from rat carotid body PGCs that activate small diameter C-fiber carotid body chemoafferents to initiate the hypoxic ventilatory response. The first set of objectives of this study was to provide direct evidence for our hypothesis, using an ultra-sensitive capacitive S-nitrosothiol sensor (Seckler et al., 2017), by determining whether 1) rat carotid body PGCs contain an S-nitrosothiol with physico-chemical properties similar to those of L-CSNO, 2) whether hypoxic challenges can stimulate isolated rat carotid body PGCs to release S-nitrosothiols, in particular L-CSNO, and 3) entry of extracellular Ca^2+^ via depolarization-induced opening of voltage-sensitive Ca^2+^-channels (Ca^2+^VS-channels) during hypoxic challenge (Donnelly and Kholwadwala, 1992; Summers et al., 2000; Kumar, 2007; Pandit and Buckler, 2009) is essential to trigger the release of S-nitrosothiols from PGCs. The second set of objectives was to provide further *in vivo* evidence to support our hypothesis by examining the ventilatory responses to intra-arterial injections of L-CSNO and L-GSNO during hypoxic gas (10% O_2_, 90% N_2_) challenge in 1) adult SHAM and CSNX rats, 2) adult rats treated as neonates with a bolus subcutaneous injection of vehicle or capsaicin in order to eliminate unmyelinated small-diameter C-fibers afferents (Meller et al., 1991), and 3) in rats receiving continuous intravenous infusions of L-SMC (10 μmol/kg/min) plus another inhibitor of L-CSNO activity, S-ethyl-L-cysteine (L-SEC, 10 μmol/kg/min) (Gaston et al., 2020).

## Methods

### Permissions

All animal studies were carried out in accordance with the National Institutes of Health Guide for the Care and Use of Laboratory Animals (NIH Publication No. 80.23) revised in 1996, and in strict compliance with the latest ARRIVE (*Animal Research: Reporting of In Vivo Experiments*) guidelines (http://www.nc3rs.org.uk/). The protocols were approved by the Institutional Animal Care and Use Committees of the University of Virginia, Case Western Reserve University, and *Galleon Pharmaceuticals, Inc*.

### Rats and surgeries

Adult male Sprague Dawley rats were obtained from Harlan Laboratories, Inc (Indianapolis, IN). The rats were caged in standard housing conditions with free access to food and water. Room temperature (22.2–22.4°C), humidity (48%–50%) and light-dark cycle (12:12 h) were maintained consistently in rooms where the rats were housed and where the studies were performed. All protocols involved the use of rats that had been implanted with an arterial catheter and/or a jugular vein catheter under isoflurane (2.5%–3.5%) anesthesia 5 days previously. To allow arterial injections of vehicle or S-nitrosothiols, the left common carotid artery was exposed and separated carefully from the sympathetic trunk and vagus nerve. Two 3.0 silk ligatures (1.5 cm apart) were placed around the common carotid artery and pulled tight to temporarily occlude blood flow (less than 60 s in total). A small hole was placed in the artery between the ligatures with a 23-gauge needle, and a non-occlusive catheter (PE-10) was inserted with the extruded tip positioned at the trifurcation of the common carotid artery where the external and internal carotid arteries split and the occipital artery branches off the external carotid artery to feed the carotid body, such that the tip of the catheter lay within 1–2 mm of the carotid body artery (Lacolley et al., 2006a; Lacolley et al., 2006b). The volumes of each intra-arterial catheter (overall mean ± SEM of 112 ± 3 μL) were measured before implantation. Following implantation, the catheter was glued in place (*Super Glue*, Elmer’s Products Inc.), and the ligatures were gradually released until full blood flow was restored. To allow for continuous infusions of L-SEC plus L-SMC, a second catheter (PE-50 connected to PE-10, the latter inserted into the vein) was inserted into the right jugular vein. All catheters were exteriorized at the back of the neck, and the wounds coated with triple antibiotic (neomycin, polymyxin B, bacitracin) ointment (*Fougera Pharmaceuticals, Inc*.) and then sutured.

### Carotid body primary glomus cell isolation

Sprague Dawley dams with litters containing 10–12 cross-fostered male pups were purchased from ENVIGO (Indianapolis, IN, United States) and delivered at least 48 h prior to experimentation. The carotid body primary glomus cell isolation procedure was adapted from Burlon et al. (2009). Briefly, three Sprague Dawley male rats (P11–16) were anesthetized with sevoflurane and decapitated. The head and neck were placed in ice cold, oxygenated (95% O_2_-5% CO_2_) Dulbecco’s phosphate buffered saline (DPBS) without Ca^2+^ or Mg^2+^. These two ions were absent from the DPBS to suppress activity of the cells. The carotid bodies were removed, cleaned of surrounding connective tissue, and transferred to a Petri dish containing digestive enzyme solution consisting of 0.4 mg/ml collagenase type I, 220 u/mg (*Worthington Biochemical Corporation, Lakewood, NJ*), and 0.2 mg/ml trypsin type I, 10,100 BAEE u/mg (*Sigma Aldrich, St. Louis, MO*) in DPBS with low CaCl_2_ (86 μM) and MgCl_2_ (350 μM) for 20 min incubation at 37 °C in a humidified, 5% CO_2_/air incubator. The carotid bodies were then mechanically teased apart and incubated for another 7 min. The tissue was then centrifuged, supernatant removed, and the cells triturated to mechanically dissociate the primary glomus cells from the remaining tissue (e.g., nerve endings, vascular endothelial cells, and type II sustentacular cells). The cells were then plated on poly-D- lysine coated glass coverslips and maintained at 37^O^C in a humidified 5% CO_2_/air incubator. The primary glomus cells were allowed to adhere for 2–3 h before use in the experiments.

### S-nitrosothiol release from carotid body primary glomus cells

Briefly coverslips were placed in a perfusion/recording chamber (RC-26GLP, Warner Instruments a division of Harvard Biosciences, Inc., Hamden, CT) and mounted on the stage of an upright microscope (BX51WI, Olympus, Shinjuku, Tokyo, Japan). The primary glomus cells were superfused with oxygenated (21% O_2_-5% CO_2_-balanced N_2_) Tyrode solution ((in mM): KCl, 4.5; NaCl, 117; CaCl_2_•2H_2_O, 2.5; MgCl_2_•6H_2_O, 1; glucose, 11; NaHCO_3_, 23, adjusted to pH 7.4 when bubbled with 5% CO_2_/air) controlled at 37 °C by an inline heater (TC-324C, Warner Instruments a division of Harvard Biosciences, Inc., Hamden, CT). For a subset of experiments the primary glomus cells were superfused with oxygenated (21% O_2_-5% CO_2_-balanced N_2_) *Ca* ^*2+*^ *-free* Tyrode solution ((in mM): KCl, 4.5; NaCl, 117; MgCl_2_•6H_2_O, 1; glucose, 11; NaHCO_3_, 23, adjusted to pH 7.4 when bubbled with 5% CO_2_/air) to assess whether L-CSNO release from carotid body primary glomus cells is via calcium-dependent vesicular fusion and exocytosis. After primary glomus cells have been superfused with oxygenated Tyrode solution, with or without calcium for 10 min a micropipette collected 200 μL of solution directly from the recording chamber (this is the normoxia group) and put it in a 500 μL Eppendorf tube for analysis by means of our capacitive biosensor (Seckler et al., 2017). Next the glomus cell perfusion was switched to a hypoxic (0%–8% O_2_-5% CO_2_-balanced N_2_) Tyrode solution, either with or without Ca^2+^, and allowed to equilibrate for 10 min before 200 μL of solution was collected directly from the recording chamber (this is the hypoxia group) and put in a 500 μL Eppendorf tube for analysis by means of our capacitive biosensor. Finally, the glomus cell perfusion was switched back to the oxygenated Tyrode solution, either with or without Ca^2+^, and allowed to equilibrate for 10 min before 200 μL of solution was collected directly from the recording chamber (this is the return group) and put it in a 500 μL Eppendorf tube for analysis by means of our capacitive biosensor.

### S-nitrosothiols stored inside of carotid body primary glomus cells

Carotid body primary glomus cells were derived from Sprague Dawley male rats (P11–16) as described above and then incubated for 10 min in DPBS with 1% (v/v) Triton X-100% and 0.5% (v/v) formaldehyde with either: nothing, 100 μM HgCl_2_, or 100 μM HgCl_2_, while being constantly exposed to UV light. This mixture was then diluted 1 to 1,000 in DPBS with 0.5% (v/v) formaldehyde and incubated for a further 5 min. The Triton X-100 served to lyse the cells, the formaldehyde served to block all free thiols and amines in the cells, and the HgCl_2_ and UV light served to degrade extant S-nitrosothiols serving as a negative control. We also measured the response generated by our system to either L-CSNO, or L-GSNO to serve as positive controls. The resulting mixture was filtered through a 10 kDa spin filter to remove membrane aggregates and large proteins, and the flow through was measured by means of our capacitive biosensor.

### Measurement of S-nitrosothiols by means of a capacitive biosensor

The levels of S-nitrosothiols in solution were measured by means of our capacitive biosensor as described previously (Seckler et al., 2017). Briefly, 3 functionalized electrodes were attached to 3 separate pre-amplifiers (SR560, Standford Research), and connected to three separate channels of an ITC-1600 (HEKA Corporation). These electrodes were houses inside of a well-ventilated Faraday cage to block out all ambient electrical noise and to ensure no exposure to formaldehyde fumes. A small Ag-AgCl ground pellet (E205, Warner Instruments) connected to a DC channel of the same ITC-1600 provided the current injection. The three electrodes were continuously perfused with DPBS with 0.5% (v/v) formaldehyde during measurements. Data was taken in 5 min intervals with a 5 min baseline, followed by sample injection and 5 min of sample wash-in, and finally 5 min of sample washout. The current response of each electrode was integrated over the duration of the current injection and that number was recorded. The difference in response between the baseline (b) and washout (w) was calculated to form a normalized ratio (r) where:

$$r=\left(w–b+\left|w–b\right|\right)/\left(2*w+2*b\right)$$

This produces a number between 0 and 1, where 0 represents all cases where the signal from the washout reading was less than or equal to the signal from the baseline reading and 1 represents the washout signal being infinitely larger than the baseline signal. In practice, any ration value greater than 0.4 represents the presence of S-nitrosothiols.

### Whole body plethysmography studies

Ventilatory parameters, frequency of breathing (Freq), tidal volume (TV) and minute ventilation (MV) were recorded in freely-moving rats by whole body plethysmography (PLY3223; Data Sciences International, St. Paul, MN) as detailed previously (May et al., 2013a; May et al., 2013b; Young et al., 2013; Getsy et al., 2014; 2020; Henderson et al., 2014; Baby et al., 2019; Gaston et al., 2020; 2021; Baby S. M. et al., 2021; Baby M. S. et al., 2021; Jenkins et al., 2021; Seckler et al., 2022). The rats were given 60 min in order to acclimatize to the chambers and to allow true resting ventilatory parameters to be established before commencing the studies.

#### SHAM and CSNX rats

These rats consisted of sham-operated (SHAM) rats and those in which both carotid sinus nerves (CSNX) were transected 7 days prior as described previously (Getsy et al., 2020; 2021a). A single arterial catheter was inserted after the transections were completed (see above for detailed description regarding arterial catheter implantation). After a 60 min acclimatization period in the plethysmography chambers, to allow the rats to settle, each carotid artery catheter was loaded with vehicle (saline), L-CSNO (300 pmol/μL) or L-GSNO (300 pmol/μL) to completely fill the catheter as per the recorded volume. A small volume (10 μL) was injected to ensure that the first test injection delivered the responses elicited by L-CSNO and L-GSNO, whereas vehicle elicited minor responses only. The doses (given as a slow bolus over 3 s) for L-CSNO were 2.5, 5, 10, 25, and 50 nmol/kg and those for L-GSNO were 5, 10, 25, 50 and 75 nmol/kg. For example, to deliver 2.5, 5, 10, 25, and 50 nmol/kg doses of L-CSNO, the volumes to be delivered would be 2.5, 5, 10, 25, and 50 μL, respectively. Each injection was given 5–10 min apart when baseline breathing values had returned to pre-injection levels for at least 90 s. The volumes given to each rat to achieve the required dose were adjusted by body weight. Equal volumes of vehicle (saline) were given to determine control (injection) responses. Other groups of adult SHAM and CSNX rats (no catheters inserted) received a 10 min hypoxic gas (10% O_2_, 90% N_2_) challenge. The dose response curves to L-CSNO and L-GSNO were designed to move through a minimal effective dose for elevating MV to one that elicits a robust response while minimally affecting mean arterial blood pressure (Gaston et al., 2020).

#### Capsaicin-treated rats

These rats had received a subcutaneous injection of vehicle (saline, 200 μL) or capsaicin (50 mg/kg) on day 3 post-birth as described previously (Meller et al., 1991). These rats received an arterial catheter as adults to give injections of vehicle or L-CSNO (2.5–50 nmol/kg, IA) or in separate rats, vehicle or L-GSNO (5–75 nmol/kg, IA). Other groups of adult vehicle- or capsaicin-treated rats (with no catheters) received a 10 min hypoxic gas (10% O_2_, 90% N_2_) challenge.

#### L-SMC + L-SEC-treated rats

These rats received a jugular vein catheter and arterial catheter as described above. The arterial catheter allowed for bolus injections of vehicle or L-CSNO (2.5–50 nmol/kg) and in other rats, vehicle or L-GSNO (5–75 nmol/kg). Groups of rats received continuous infusions of vehicle (20 μL/min) or L-SMC (10 μmol/kg/min) plus L-SEC (10 μmol/kg/min). Other groups of rats receiving vehicle or L-SMC (10 μmol/kg/min) plus L-SEC (10 μmol/kg/min) were exposed to a 10 min hypoxic gas (10% O_2_, 90% N_2_) challenge beginning 30–35 min after starting the infusions (30 min was a sufficient amount of time for ventilatory parameters to stabilize).

### Data analyses

All data are presented as mean ± SEM and were evaluated using one-way and two-way ANOVA followed by Bonferroni corrections for multiple comparisons between means using the error mean square terms from each ANOVA analysis (Winer, 1971; Wallenstein et al., 1980; Ludbrook, 1998; McHugh, 2011) as detailed previously (Getsy et al., 2021a; Jenkins et al., 2021). A value of *p* < 0.05 was taken as the initial level of statistical significance (Wallenstein et al., 1980; Ludbrook, 1998; McHugh, 2011). The statistical analyses were performed using GraphPad Prism software (GraphPad Software, Inc., La Jolla, CA). A detailed description of these statistical procedures is provided in the Supplementary Material (see *Detailed description of Statistical Approaches*).

## Results

**FIGURE 1:**
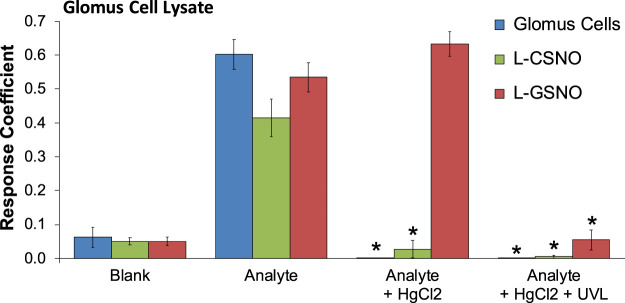
The response coefficients of our S-nitrosothiol biosensor to a 1,000x dilution of primary glomus cell lysates (analyte), 100 fM S-nitroso-L-cysteine (L-CSNO) or 100 fM S-nitrosoglutathione (L-GSNO). Definitions: Columns designated as “Blank” refer to the electrode reaction to Dulbecco’s phosphate buffered saline (DPBS) alone for each study. Columns designated as “Analyte” refer to the electrode reaction to primary glomus cell lysates (Glomus cells), L-CSNO (100 fM) or L-GSNO (100 fM). The columns designated as “Analyte + HgCl_2_” refer to the electrode reactions to primary glomus cell lysates (Glomus cells), L-CSNO (100 fM) or L-GSNO (100 fM) that were exposed to HgCl_2_ (100 μM). The columns designated as “Analyte + HgCl_2_ + UVL” refer to the electrode reactions to primary glomus cell lysates (Glomus cells), L-CSNO (100 fM) or L-GSNO (100 fM) that were exposed to HgCl_2_ (100 μM) and ultraviolet light (UVL). The data are shown as mean ± SEM from 6-7 samples. *p < 0.05, significant change from control conditions (Analyte columns).

**FIGURE 2:**
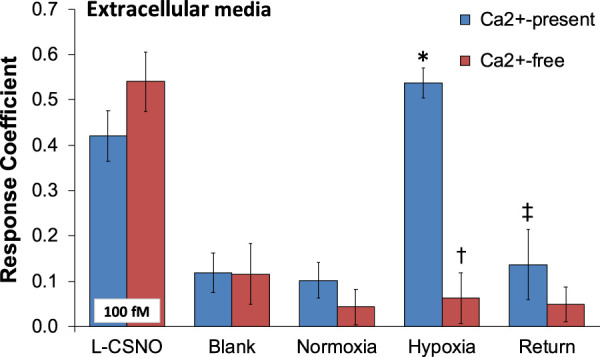
The response coefficient of the S-nitrosothiol biosensors to perfusion media collected from the chamber of plated PGCs exposed to normoxia, hypoxia, or return to normoxia after hypoxic challenge. All of the electrodes were exposed to DPBS before the experiment (blank) to serve as a negative control. After each experiment, electrodes were exposed to a solution of 100 fM S-nitroso-L-cysteine (L-CSNO) to ensure that the electrodes were properly detecting S-nitrosothiols and specifically, L-CSNO. The data are presented as mean ± SEM. There were 6 samples in each of the Ca^2+^-present studies and 5 samples in each of the Ca^2+^-absent studies. *p < 0.05, significant difference from blank and normoxia values. ^†^ *p* < 0.05, Ca^2+^-free versus Ca^2+^-values resulting from hypoxic challenge. ‡*p* < 0.05, Ca^2+^-free versus Ca^2+^-values upon return to room-air.

As can be seen in Figure 1, primary glomus cell lysates contained S-nitrosothiol concentrations equivalent to 100 fM concentrations of L-CSNO and 100 fM concentrations of L-GSNO. The addition of HgCl_2_ (100 μM) abolished the S-nitrosothiol signal from the primary glomus cell lysate and authentic L-CSNO (100 fM) but not that of L-GSNO. The S-nitrosothiol signals from the primary glomus cell lysate, L-CSNO (100 fM) and GSNO (100 fM) were effectively abolished by co-application of HgCl_2_ (100 μM) and UV light. Additionally, DPBS was used as a negative control (blanks). As can be seen in Figure 2, no significant S-nitrosothiol signal above blank (DPBS) was observed in the extracellular media under normoxic conditions (21% O_2_-5% CO_2_-balanced N_2_). After 10 min exposure to hypoxia ((0%–8% O_2_-5% CO_2_-balanced N_2_), the concentrations of S-nitrosothiols in the extracellular media containing Ca^2+^ reached peak levels similar to those produced by a 100 fM concentration of authentic L-CSNO, and no significant level of S-nitrosothiols was detected in the Ca^2+^-free media. After 10 min return to normoxia the S-nitrosothiol levels returned to the pre-hypoxic levels in the extracellular media containing Ca^2+^. These results show that S-nitrosothiols release from carotid body glomus cells is calcium-dependent and most likely involves calcium-dependent vesicular fusion and release.

### *In vivo* studies - Rat descriptors and resting ventilatory parameters

The body weights and number of rats in each group are summarized in Supplementary Table S1 (there were no between-group differences in body weights for any study (*p* > 0.05, for all comparisons). The actual Freq, TV and MV data are summarized in Supplementary Table S2-S5. The data show that i) resting ventilatory parameters in the CSNX groups were generally lower than those in the SHAM groups, ii) resting parameters were similar in the VEH and CAP groups and iii) resting parameters in the groups of rats receiving infusion of L-SMC + L-SEC were generally lower than those receiving infusions of vehicle. In addition, Supplementary Table S6 shows that the infusion of L-SMC + L-SEC caused sustained decreases in Freq, TV and MV from initial control values, whereas ventilatory values remained at pre-infusion values in rats receiving vehicle (saline).

### Hypoxic ventilatory responses

**FIGURE 3:**
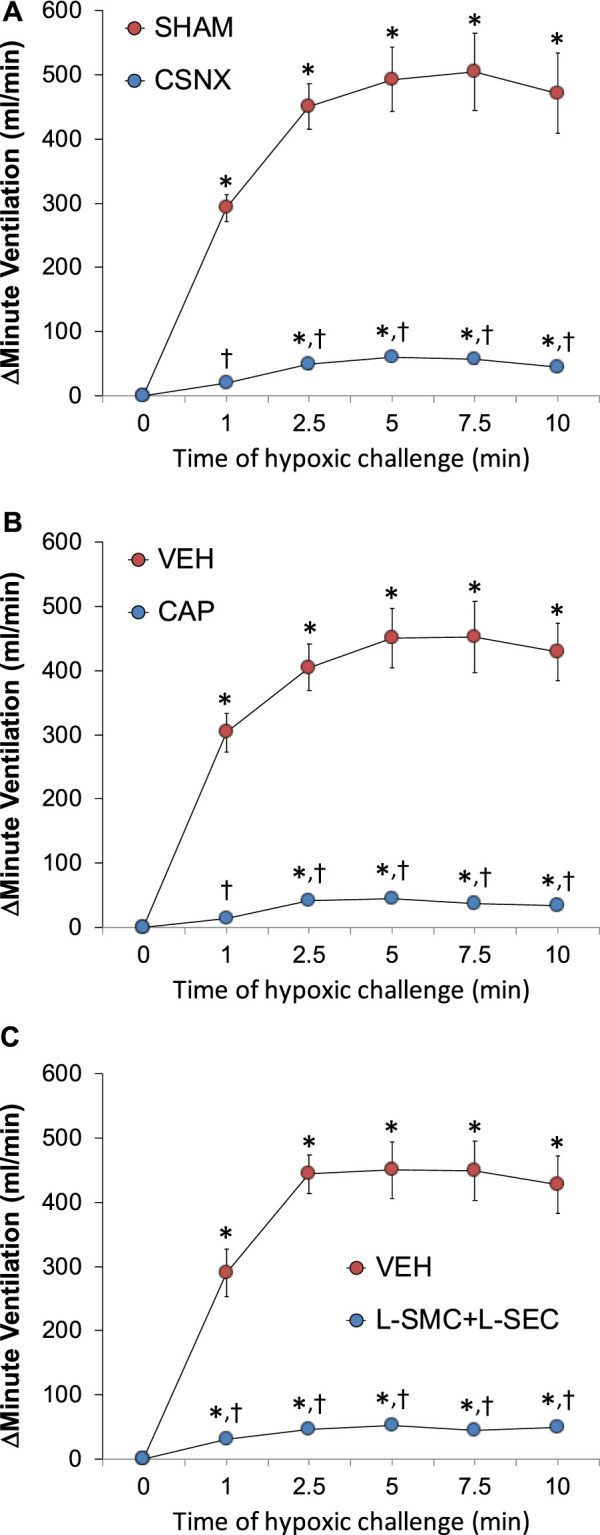
Arithmetic changes in minute ventilation during a hypoxic gas challenge (10% O_2_, 90% N_2_) for 10 min. **(A)** Adult sham-operated (SHAM) rats (n = 6) and those with bilateral carotid sinus nerve transection (CSNX) (n = 6). **(B)** Adult rats treated as neonates with vehicle (VEH; n = 9) or capsaicin (CAP; 50 mg/kg, SC; n = 9). **(C)** Adult rats receiving a continuous intravenous infusion of vehicle (VEH; 20 μL/min, IV; n = 9) or S-methyl-L-cysteine (L-SMC; 10 μmol/min, IV; n = 9) plus S-ethyl-L-cysteine (L-SEC; 10 μmol/min, IV; n = 9). The data are shown as mean ± SEM. *p < 0.05, significant response. †*p* < 0.05, CSNX versus SHAM, CAP versus VEH, L-SMC + L-SEC versus VEH.

As can be seen in Panel A of Figure 3, exposure to a hypoxic gas (10% O_2_, 90% N_2_) challenge for 10 min elicited a robust increase in MV in sham-operated (SHAM rats) and markedly smaller responses in bilateral CSN-transected (CSNX) rats. Similar patterns of responses were observed for Freq and TV (Supplementary Figure S1, Panels A and D). As seen in Panel B of Figure 3, exposure to a hypoxic gas (10% O_2_, 90% N_2_) challenge for 10 min elicited a robust increase in MV in neonatal vehicle-injected (VEH) rats and markedly smaller responses in neonatal capsaicin-injected (CAP) rats. Similar patterns of responses were observed for Freq and TV (Supplementary Figure S1, Panels B and E). Moreover, as seen in Panel C of Figure 3, exposure to a hypoxic gas (10% O_2_, 90% N_2_) challenge for 10 min elicited a robust increase in MV in rats receiving an intravenous infusion of vehicle (VEH) rats and markedly smaller responses in those receiving an infusion of L-SMC + L-SEC. Similar patterns of responses were observed for Freq and TV (Supplementary Figure S1, Panels C and F).

### S-nitrosothiol responses

**FIGURE 4:**
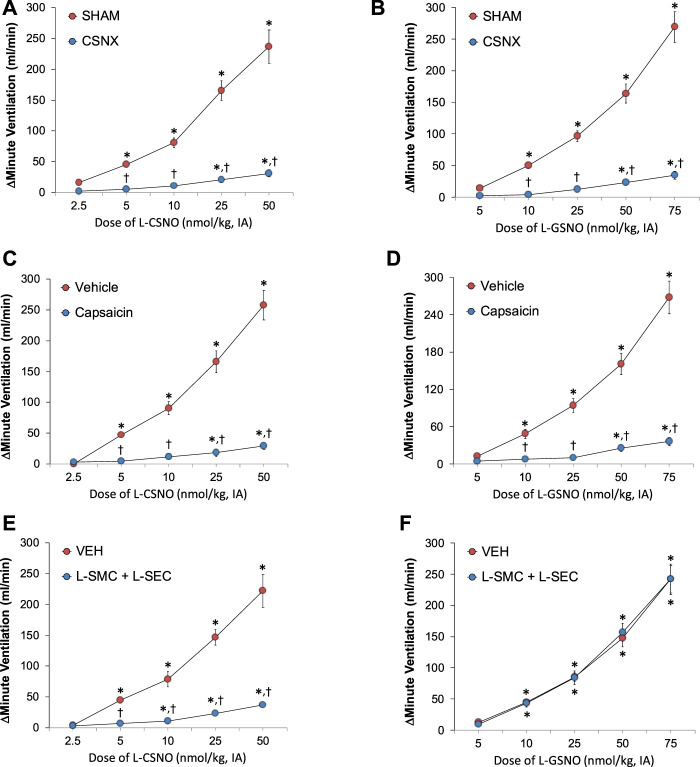
Arithmetic changes in minute ventilation elicited by injections of L-CSNO (left-hand panels) and L-GSNO (right-hand panels). **(A)** and **(B)** Adult sham-operated (SHAM) rats (n = 6) and those with bilateral carotid sinus nerve transection (CSNX) (n = 6). **(C)** and **(D)** Adult rats treated as neonates with vehicle (VEH; n = 9) or capsaicin (CAP; 50 mg/kg, SC; n = 9). **(E)** and **(F)** Adult rats receiving a continuous intravenous infusion of vehicle (VEH; 20 μL/min, IV; n = 9) or S-methyl-L-cysteine (L-SMC; 10 μmol/min, IV; n = 9) plus S-ethyl-L-cysteine (L-SEC; 10 μmol/min, IV; n = 9). The data are shown as mean ± SEM. *p < 0.05, significant response. †*p* < 0.05, CSNX versus SHAM, CAP versus VEH, L-SMC + L-SEC versus VEH.

As can be seen in panels A and B of Figure 4, respectively, intra-arterial injections of L-CSNO (2.5–50 nmol/kg) or L-GSNO (5–75 nmol/kg) elicited dose-dependent increases in MV in SHAM rats and markedly smaller responses in CSNX rats. As seen in panels C and D of Figure 4, respectively, these injections of L-CSNO or L-GSNO elicited dose-dependent increases in MV in VEH rats and markedly smaller responses in CAP rats. As seen in panel E of Figure 4, the injections of L-CSNO elicited dose-dependent increases in MV in rats receiving VEH infusion and markedly smaller responses in rats receiving an infusion of L-SMC + L-SEC. In contrast, the dose-dependent increases in MV elicited by L-GSNO in the L-SMC + L-SEC rats were similar to those in VEH rats (Figure 4F,). Similar patterns of responses were observed for Freq and TV for L-CSNO (Supplementary Figure S2) and for L-GSNO (Supplementary Figure S3) in all three groups.

## Discussion

Capacitive sensors when coupled to a semiconducting material represent a method to detect trace quantities of a chemical in complex solutions. We have taken advantage of the semiconducting and chemical properties of polydopamine to construct a capacitive sensor, which specifically senses S-nitrosothiols in complex biological solutions (Seckler et al., 2020). Our capacitive biosensor employs a thin layer of polydopamine, which will crosslink to all free amines, free thiols, and S-nitrosothiols within the body. To make our sensor selective for small molecule S-nitrosothiols we used the following process: 1) We ran our sample through a 10 kDa spin filter to remove all large proteins and other molecules, 2) We incubated our sample in 0.4% formaldehyde to block all free thiols and free amines, but not S-nitrosothiols, 3) We divided this sample into three aliquots and performed 2 separate negative controls 4) We pre-incubated one negative control to HgCl_2_, and 5) We exposed the other negative control to UV light with an emission of 350 nm. These steps ensure that we selectively sense S-nitrosothiols. If our signal, for example, instead came from a small molecule which can bind to polydopamine, but cannot be fully blocked by formaldehyde, such as urea, then steps 4 and 5 would fail as HgCl_2_ and/or UV light would not be capable of abolishing a signal. Additionally, if our signal was produced by a small molecule, such as cysteine sulfinic acid, it would show a signal when exposed to UV light (as our UV does not emit in the 300 nm absorption band of sulfinic acid), but not in HgCl_2_ (which is capable of degrading sulfinic acid). Therefore, the method by which the capacitive biosensor studies were performed left no alternative targets for the biosensor apart from small molecule S-nitrosothiols.

The first major finding of the present study was that rat PGCs contained an S-nitrosothiol with physico-chemical properties more closely resembling those of L-CSNO than L-GSNO. The presence of pre-formed S-nitrosothiols in intracellular vesicles of PGCs is not shocking as there is published evidence showing the presence of S-nitrosothiols in cytoplasmic vesicles of endothelial cells (Seckler et al., 2020). There is also direct evidence that rat PGCs contain vesicles that store neurotransmitters, such as catecholamines (e.g., dopamine) and ATP that are subject to depolarization-induced (Weiss and Donnelly, 1996), Ca^2+^-*dependent* exocytosis in response to stimuli, including hypoxia (Echeverría et al., 1977; 1978; Hansen, 1977; Grönblad and Eränkö, 1978; Krammer, 1978; Grönblad et al., 1979; 1980; Grönblad, 1983; Conde et al., 2009; Yan et al., 2012; Ortega-Sáenz et al., 2016; Tse et al., 2018) via the presence of VGCa-channels (Silva and Lewis, 1995; Peers and Buckler, 1995; Overholt and Prabhakar, 1997; Carpenter et al., 2000; Peers, 2004; Makarenko et al., 2015; Conde et al., 2006) and Ca^2+^-binding sites (Hansen and Smith, 1979) in the plasma membranes of PGCs. These observations are clinically relevant since human PGCs contain large numbers of cytoplasmic vesicles (Abrahám, 1970; 1981; Kobayashi, 1971a; Kobayashi, 1971b; Hervonen and Korkala, 1972) that contain gap-junctions (Kondo, 2002), enkephalin (Khan, et al., 1990) and numerous other peptides/polypeptides (Smith et al., 1990; Scraggs et al., 1992), such as calcitonin, calcitonin gene-related peptide, cholecystokinin and Substance P (Kummer and Habeck, 1991; Wang, et al., 1993). The question arises as to how S-nitrosothiols, such as L-CSNO, may be stored in vesicles subject to exocytosis. Neuronal and endothelial forms of nitric oxide synthase (nNOS and eNOS, respectively) are expressed in several structures in the carotid bodies, including sensory nerve terminals, ganglion cells and vascular endothelium (Prabhakar et al., 1993; Wang et al., 1993; Grimes et al., 1994; 1995; Höhler et al., 1994; Atanasova et al., 2016; 2020). Importantly, while several studies have not found eNOS or nNOS to be expressed in carotid body primary or secondary (sustenacular) glomus cells (Prabhakar et al., 1993; Höhler et al., 1994; Grimes et al., 1995; Moya et al., 2012; Atanasova et al., 2016; 2020), others have demonstrated the presence of eNOS in rat (Yamamoto et al., 2006) and cat (Valdés et al., 2003) PGCs and nNOS in rabbit PGCs (Li et al., 2010). Moreover, Grimes et al. (1994) observed weak NADPH diaphorase staining in rat PGCs and Tanaka and Chiba (1994) demonstrated intense NADPH diaphorase staining in sub-populations of guinea-pig PGCs. Although Atanasova et al. (2020) reported that PGCs were largely negative for NADPH diaphorase, close analysis of their figures shows that it is present in fine granules in the glomus cells, but not as robust as in the encircling fine neural positive varicosities. These findings are relevant because of our evidence that NADPH diaphorase is a histochemical marker for S-nitrosylated proteins and small molecular weight S-nitrosothiols (Seckler et al., 2020). With respect to how S-nitrosothiols may be generated in PGCs, there is substantial evidence that plasma membranes of intracellular vesicles in other structures, such as nerve terminals and endothelial cells, contain NOS (Loesch et al., 1993; 1994; Dikranian et al., 1994; Loesch and Burnstock, 1995; Sosunov et al., 1995; Loesch and Burnstock, 1996; Sosunov et al., 1996; Loesch and Burnstock, 1998), whereas NADPH diaphorase is localized strictly to the lumen of these vesicles (Loesch et al., 1993). Our evidence that NADPH diaphorase detects S-nitrosothiols/S-nitrosylated proteins (e.g., NOS) led us to determine that cytoplasmic vesicles extracted from rat femoral artery endothelium are indeed rich in S-nitrosothiols (Seckler et al., 2020). Moreover, the presence of cysteine transporters, such as excitatory amino acid transporters 1 and 3 on PGC membranes of rats and humans (Li et al., 2020), would allow for entry of L-cysteine into PGCs for potential transformation to L-CSNO.

The second major finding of this study was that hypoxic challenge caused the release of S-nitrosothiols (presumably L-CSNO) from these PGCs that was strictly dependent on the presence of extracellular Ca^2+^. The question arises as to whether the hypoxia-stimulated Ca^2+^-*dependent* release of L-CSNO from the carotid body PGCs is from *de novo* synthesized L-CSNO or potentially from pre-formed vesicular pools that are subject to exocytosis. L-CSNO is poorly lipophilic (Clancy et al., 2001) and its *entry* into cells occurs predominantly/exclusively via L-amino acid transporter systems (Li et al., 2020; Nemoto et al., 2003; Li and Whorton, 2007). Although to our knowledge there is no evidence that L-CSNO can be transported out of cells via these L-amino acid transporter systems, it may be possible that hypoxia elicits a Ca^2+^-*dependent* increase in L-CSNO synthesis within PGCs that then undergo transport to the extracellular environment. It would also seem plausible that hypoxia elicits Ca^2+^-dependent exocytosis of vesicular pools of L-CSNO. To our knowledge there is no evidence that the plasma membranes of PGCs or vesicles within PGCs contain fusion proteins that are known to subserve exocytosis on other cell-types (Sudhof, 2004; Südhof and Rothman, 2009; Wang et al., 2019). Nonetheless, such a process would allow for dynamic regulation of carotid body chemoafferent activity in response to graded levels of hypoxia and presumably hypercapnia and acidosis. It would also seem plausible that these putative stores of L-CSNO may be depleted upon repetitive hypoxic gas challenges when NOS in PGC is inhibited, thereby preventing the replenishing of these L-CSNO stores resulting in progressively smaller ventilatory responses. Such use-dependent loss of vasodilation in the presence of NOS inhibitors has been observed in response to repetitive stimulation of the vascular endothelium (Davisson et al., Batenburg et al., 2004a; b, 2009) and lumbar sympathetic fibers (Davisson et al., 1996b; Davisson et al., 1996c; Davisson et al., 1997a). Indeed, Gozal et al. (1985) reported the hypoxic ventilatory response in freely-moving rats rapidly declines in the presence of a non-specific inhibitor of eNOS and nNOS or a specific inhibitor of nNOS.

The third major finding was that the ventilatory responses elicited by L-CSNO and a hypoxic gas challenge in freely-moving adult Sprague Dawley rats were markedly diminished in rats receiving a continuous infusion of L-SMC + L-SEC. The observation that the ventilatory responses to L-GSNO were not diminished by the L-SMC + L-SEC infusion provides support for the above biochemical findings that rat PGCs store L-CSNO and release it response to hypoxic challenge. We have previously reported that the responses to hypoxia and L-CSNO were diminished in rats receiving L-SMC alone, but we wanted to further our studies to include L-SEC, since our preliminary cardiovascular studies are showing that L-SEC is a better inhibitor than L-SMC in some vascular beds (Lewis, unpublished observations). Taken with the other *in vivo* studies presented here (CSNX and CAP studies) it seems that hypoxia may release L-CSNO from PGCs that in turn activate small diameter unmyelinated C-fiber chemoafferents to mediate the HVR. The hypoxic ventilatory responses recorded in this study are consistent with evidence that the ventilatory responses to hypoxic gas challenges are largely abolished in adult rats with CSNX (Getsy et al., 2020; 2021a) and adult rats treated with capsaicin as neonates (McQueen and Mir, 1984; De Sanctis et al., 1991). Our data that the ventilatory responses elicited by L-CSNO and L-GSNO are markedly diminished in CSNX and CAP rats greatly expands our understanding of the pharmacology of these endogenous S-nitrosothiols. It should be noted that the infusion of L-SEC + L-SEC caused a reduction in resting frequency and tidal volume, and therefore minute ventilation in naïve rats. The decreases in these parameters could be due to numerous effects, such as sedation (not observed to be the case) or a decrease in resting carotid body chemoafferent activity via inhibition of the possible tonic effects of PGC-released L-CSNO that may occur under normoxic conditions. The decreases in ventilatory parameters appear to be consistent with those that occur during application of the Dejours’ test (100% oxygen), which is known to silence carotid body chemoafferents (Haouzi et al., 2014).

## Study limitations

A study limitation is that although the signals from the PGC lysates exposed to HgCl_2_ and UV light showed a degradation pattern consistent with the signals being due to the presence of L-CSNO, but not L-GSNO, it remains possible that the S-nitrosothiol present in PGCs is another small molecule S-nitrosothiol with a free primary amine, such as S-nitroso-cysteamine, S-nitroso-coenzyme A, or S-nitroso-glutamylcysteine (for which there is no evidence currently for their existence in PGCs). The development of a selective antibody to L-CSNO would allow immunohistochemical studies to confirm the presence of L-CSNO in PGCs. Another study limitation is that the S-nitrosothiol detection experiments were performed in PGCs from P11-P16 rat pups and not in cells from adult rats that were used for the whole body plethysmography studies. PGCs from rat pups were used 1) because it is extremely difficult to isolate PGCs from adult carotid bodies due to extensive connective tissue and other technical matters, and 2) to match on-going electrophysiological whole cell patch-clamp recordings that are examining the effects of L-CSNO on carotid body PGC responses to hypoxia that are historically only performed successfully in young rat pups (P10-P25). Another limitation pertains to the lack of S-nitrosothiol detection studies in PGCs from female rats and plethsmography studies in freely-moving female rats since it is well-established that sex differences occur in terms of specific patterns of changes in breathing parameters and cell-signaling events in response to hypoxic and hypercapnic gas challenges (Getsy et al., 2021b; Getsy et al., 2021c). Additionally, our study is limited in that only Sprague Dawley rats were used and it would be important to extend our studies to include other strains of rats and also larger species to get a more complete picture of the potential role of PGC-derived S-nitrosothiols in the carotid body control of ventilation. Finally, dose-response studies with L-SMC + L-SEC would also further the pharmacological analyses regarding the role of PGC-derived L-CSNO in ventilatory control processes. The intra-arterial doses of L-CSNO used in these studies (2.5–50 nmol/kg) elicited increases in MV that were substantially less in magnitude than those elicited by the hypoxic gas challenge. These doses were chosen because they elicit minimal falls in arterial blood pressure that may confound the interpretation of the MV responses (Gaston et al., 2020). Although the present findings raise the distinct possibility that primary glomus cell-derived L-CSNO has a possible role in the expression of the hypoxic ventilatory response (HVR), we have preliminary evidence that high doses of L-CSNO (e.g., 100 nmol/kg, IA) elicit a rise in MV of magnitude closely matching that of the hypoxic gas challenge. Although the combination of L-SMC + L-SEC markedly diminished the responses to L-CSNO and hypoxic gas challenge, there were certainly residual responses in the order of 50 ml/min in both the hypoxic gas challenge and the 50 nmol/kg dose of L-CSNO. At present we do not know whether higher doses of L-SMC + L-SEC would elicit a more complete blockade of the L-CSNO responses, or whether L-S-phenyl-cysteine (L-SPC), which we used in combination with L-SMC to identify L-CSNO interacting proteins (Gaston et al., 2020), would in combination with L-SMC and L-SEC provide a more complete blockade of the effects of L-CSNO and hypoxia on MV. The tentative idea driving the use of various combinations of L-SMC, L-SEC and L-SPC is that they may differentially block the numerous functional proteins (e.g., K_v_-channels) activated by L-CSNO (Gaston et al., 2020).

## Conclusion

**FIGURE 5:**
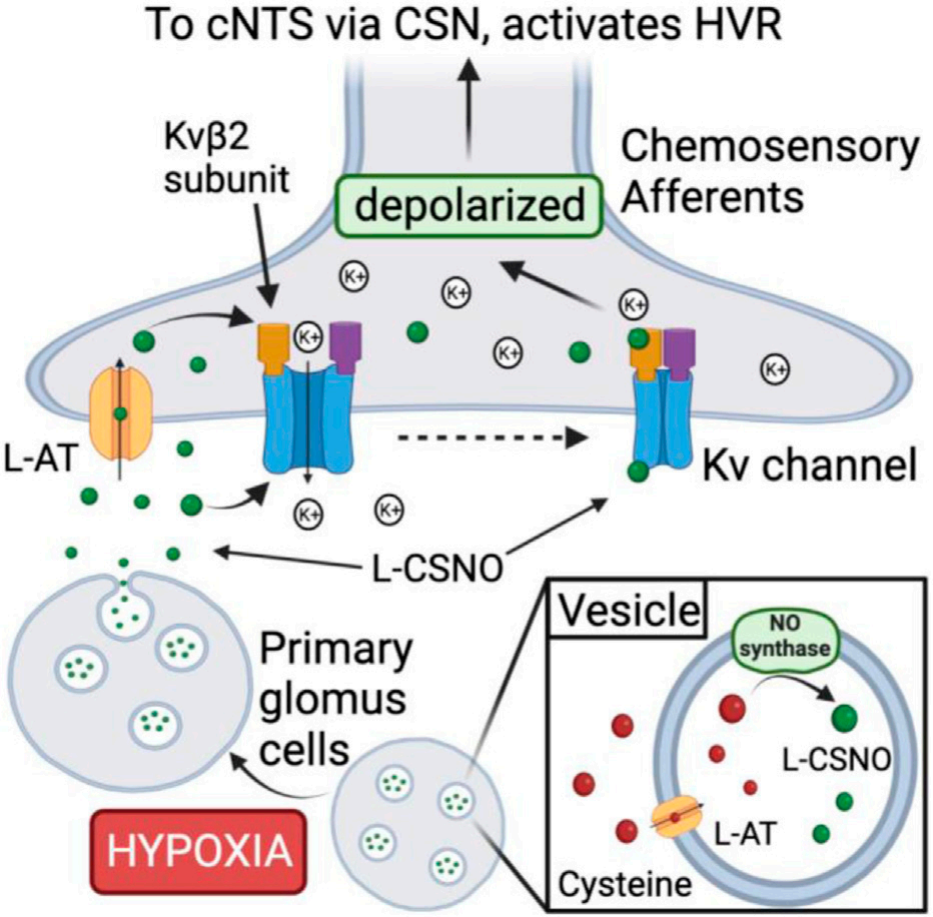
Proposed mechanism by which S-nitroso-L-cysteine (L-CSNO) is stored in primary glomus cells (PGCs) and released in response to hypoxia to act as a neurotransmitter mediating the hypoxic ventilatory response. Vesicular stores of L-CSNO within PGCs are generated by L-cysteine uptake into vesicles via membrane-bound L-amino acid transporter (L-AT) and then S-nitrosylation of L-cysteine to L-CSNO by membrane-bound nitric oxide synthase (NOS). Hypoxia causes PGCs to depolarize thereby activating voltage-gated Ca^2+^-channels (VGCa-channels) leading to extracellular Ca^2+^-*dependent* release of the vesicular stores of L-CSNO. The released L-CSNO then binds stereoselectively to extracellular domains of voltage-gated K^+^-channels (Kv-channels), such as Kvβ2 subunits, on carotid body chemoafferent nerve terminals to actively close the Kv-channels thereby preventing K^+^ release from the terminals. Diminished K^+^ release depolarizes the terminals thereby generating action potentials that activate neurons in the commissural nucleus tractus solitarius (cNTS) leading to neurotransmitter release that elicits the hypoxic ventilatory response (HVR). In addition, L-CSNO released by the PGCs is actively transported into chemoafferent terminals in the carotid body via plasma membrane-bound L-AT.

As presented in Figure 5, our major conclusions are that carotid body PGCs from male Sprague Dawley rats contain an S-nitrosothiol with physico-chemical properties resembling those of L-CSNO, and that a hypoxic gas challenge releases L-CSNO in a manner dependent upon Ca^2+^-entry into PGCs. Our studies with L-SMC + L-SEC support the concept that the PGC-derived S-nitrosothiol is L-CSNO, since the ventilatory actions of L-CSNO and hypoxic gas challenge were markedly attenuated by L-SMC + L-SEC, whereas the responses elicited by L-GSNO were not. Accordingly, our data support the concept that the release of L-CSNO from PGCs plays a primary role in the expression of the HVR (Figure 5). Whether PGC-derived L-CSNO directly excites carotid body afferents and/or releases excitatory neurotransmitters from PGCs is undetermined. Whether L-CSNO contributes to the ventilatory responses that occur post-hypoxic (Getsy et al., 2021b) and post-hypercapnic (Getsy et al., 2021c) challenges, and which post-response depends upon carotid body chemoafferents (Getsy et al., 2020; Getsy et al., 2021a), is also undetermined. Other possible mechanisms of action for L-CSNO may include a) L-CSNO-stimulated release of neurotransmitters from primary glomus cells, which activate chemoafferents, and/or b) L-CSNO-mediated S-nitrosylation of functional proteins on the chemoafferent terminals thereby allowing other neurotransmitters to directly activate the afferents. While the ventilatory responses elicited by IA injections of L-CSNO appear to involve the direct/indirect activation of carotid body chemoafferents, the residual responses observed for the higher doses of L-CSNO in the CSNX and CAP rats raises the possibility that L-CSNO may exert its effects via actions on other afferent fibers or at brain sites that are devoid of a blood-brain barrier, such as the area postrema (Johnson and Gross, 1993). The ability of glomus cell-derived L-CSNO to activate the carotid body chemoreflex may be part of a coordinated role of this S-nitrosothiol in regulating cardio-respiratory function at the brainstem level. More specifically, we reported that the NADPH diaphorase technique visualizes S-nitrosothiols/S-nitrosylated proteins in the brain (Seckler et al., 2020), and it is evident that afferent fibers entering the nucleus tractus solitarius (NTS) and numerous intrinsic cells within the NTS stain for NADPH diaphorase (Ruggiero et al., 1996). This and the ability of microinjections of L-CSNO into the NTS of rats to lower mean arterial blood pressure (Ohta et al., 1997) provide tentative support for the concept that primary afferent terminals or intrinsic neurons release L-CSNO or related S-nitrosothiols as neurotransmitters/neuromulators to drive cardiovascular and potentially ventilatory response processes within the NTS. The possibility that the loss of afferent input to the NTS in CSNX or CAP rats alters the responsiveness of NTS neurons to intrinsic L-CSNO is an intriguing question.

## Data Availability

The raw data supporting the conclusion of this article will be made available by the authors, without undue reservation.

## Abbreviations

PGCs, primary glomus cells; L-CSNO, S-nitroso-L-cysteine; L-GSNO, L-S-nitrosoglutathione; L-SMC, S-methyl-L-cysteine; L-SEC, S-ethyl-L-cysteine; Freq, frequency of breathing; TV, tidal volume; MV, minute ventilation; Kv-channels, voltage-gated K + -channels; HgCl2, mercurous chloride; UV light, ultra-violet light; CSNX, carotid sinus nerve transection; CAP, capsaicin; VEH, vehicle.
