# Supplementary material for: Hypoxia releases S-nitrosocysteine from carotid body glomus cells—relevance to expression of the hypoxic ventilatory response
Source: Front Pharmacol. 2023 Oct 11;14:1250154. doi: 10.3389/fphar.2023.1250154 (PMC10598756; doi:10.3389/fphar.2023.1250154)
Supplement: Supplementary file 1 [file DataSheet1.docx]

**Supplementary File**

**Hypoxia releases S-nitrosocysteine from carotid body glomus cells – relevance to expression of the hypoxic ventilatory response**

James M. Seckler,^1^ Paulina M. Getsy,^2^ Walter J. May,^3^ Benjamin Gaston,^4^

Santhosh M. Baby,^5^ Tristan H.J. Lewis,^2^ James N. Bates,^6,†^ Stephen J. Lewis^2,7,8,^*

*^1^Department of Biomedical Engineering, Case Western Reserve University,*

*Cleveland, Ohio, USA*

*^2^Departments of Pediatrics, Case Western Reserve University, Cleveland, Ohio, USA*

*^3^Department of Pediatrics, University of Virginia, Charlottesville, Virginia, USA*

*^4^Herman B Wells Center for Pediatric Research, Indiana University School of Medicine,*

*Indianapolis, Indiana, USA*

*^5^Galleon Pharmaceuticals, Inc., 213 Witmer Road, Horsham, PA, USA*

*^6^Department of Anesthesia, University of Iowa, Iowa City, IA, USA*

*^7^Departments of Pharmacology, Case Western Reserve University, Cleveland, OH, USA*

*^8^Functional Electrical Stimulation Center, Case Western Reserve University, Cleveland, OH, USA*

**^†^Current address:** James N. Bates, Chief Medical Officer, *Atelerix Life Sciences Inc*. Address: 300 East Main Street, Suite 202 Charlottesville, Virginia 22902. Email: jbates@atelerixlifesciences.com. https://atelerixlifesciences.com

***Corresponding Author:** Stephen J. Lewis, PhD. Department of Pediatrics, Division of Pulmonology, Allergy and Immunology, Department of Pharmacology School of Medicine, Biomedical Research Building, Room 831, Case Western Reserve University, 10900 Euclid Avenue, Cleveland, OH 44106-4984. Phone: 843-422-7639. Email: sjl78@case.edu

**^†^Current address:** Santhosh M. Baby, Translational Sciences Treatment Discovery, Galvani Bioelectronics, Inc., 1250 S Collegeville Rd., Collegeville, Pennsylvania 19426. Email: santhosh.m.baby@galvani.bio

**Detailed description of Statistical Approaches**

The recorded data (1 min bins), derived parameters, and response areas (cumulative percent changes from designated pre-values) were taken for statistical analyses. The pre-drug 1 min bins excluded the occasional marked deviations from resting due to erratic movements of the rats, such as scratching. The exclusions ensured accurate determinations of baseline parameters. The data are presented as mean ± SEM. All of the data were analyzed by one-way or two-way analysis of variance followed by Student’s modified t-test with Bonferroni corrections for multiple comparisons between means employing the modified error mean square term (EMS) from the ANOVA (Wallenstein et al., 1980). The modified t-statistic is t = (mean group 1- mean group 2)/[s x (1/n1 + 1/n2)^1/2^] where s^2^ = the mean square within groups term from the ANOVA (the square root is taken for the modified t-statistic formula) and n1 and n2 are the numbers of the rats in each group. Based on an elementary inequality called Bonferroni’s inequality, a conservative critical value for the modified t-statistics were obtained from tables of the t-distribution using a significance level of P/m, where m is the number of comparisons between groups to be performed. The degrees of freedom are those for the mean square for the within group variation from the ANOVA table. In most cases, the critical Bonferroni value cannot simply be obtained from conventional tables of the t- distribution but may be approximated from widely available tables of the normal curve by t* = z + (z + z3)/4n, where n is the degrees of freedom and z is the critical normal curve value for P/m (Winer, 1971). As demonstrated clearly by Wallenstein et al (1980), the Bonferroni procedure is recommended for general use since it (a) is the easiest to apply, (b) has the widest range of applications, (c) gives critical values lower than those of other procedures if the investigator is able to limit the number of comparisons, and (d) gives critical values that will be only slightly larger than those of other procedures if many comparisons are made. A value of *p* < 0.05 was taken as the initial level of statistical significance (Winer, 1971; Wallenstein et al., 1980).

**References for text**

Wallenstein, S., Zucker, C.L., and Fleiss, J.L. (1980). Some statistical methods useful in circulation research. *Circ. Res*. 47, 1-9. doi: 10.1161/01.res.47.1.1.

Winer, B.J. (1971). Statistical principles of experimental design. McGraw-Hill Book Co., pp 752-809.

**Supplementary Table S1.** Number and body weights of rats in SHAM and CSNX groups for all studies

|  |  |  |  | **Parameter** | | |
| --- | --- | --- | --- | --- | --- | --- |
| **Study** |  | **Groups** |  | **Rat Numbers** |  | **Body Weight, g** |
| **Hypoxia Study** |  | SHAM |  | 9 |  | 334 ± 1.4 |
|  |  | CSNX |  | 9 |  | 331 ± 1.6 |
|  |  | VEH |  | 6 |  | 335 ± 2.1 |
|  |  | CAP |  | 6 |  | 336 ± 2.5 |
|  |  | VEH |  | 9 |  | 333 ± 1.8 |
|  |  | L-SMC + L-SEC |  | 9 |  | 334 ± 1.7 |
| **S-nitroso-L-cysteine Study** |  | SHAM |  | 9 |  | 335 ± 1.8 |
|  |  | CSNX |  | 9 |  | 332 ± 2.5 |
|  |  | VEH |  | 9 |  | 333 ± 2.2 |
|  |  | CAP |  | 9 |  | 334 ± 2.1 |
|  |  | VEH |  | 9 |  | 333 ± 1.8 |
|  |  | L-SMC + L-SEC |  | 9 |  | 331 ± 2.9 |
| **S-nitroso-L-glutathione Study** |  | SHAM |  | 9 |  | 331 ± 2.0 |
|  |  | CSNX |  | 9 |  | 333 ± 1.8 |
|  |  | VEH |  | 9 |  | 334 ± 1.8 |
|  |  | CAP |  | 9 |  | 331 ± 2.8 |
|  |  | VEH |  | 9 |  | 335 ± 1.6 |
|  |  | L-SMC + L-SEC |  | 9 |  | 336 ± 1.4 |

SHAM, sham-operated rats; CSNX, rats with bilateral carotid sinus nerve transection; VEH, vehicle-treated rats; CAP, capsaicin-treated rats; L-SMC + L-SEC, rats that were receiving co-infusions of S-methyl-L-cysteine (10 μmol/kg, IV) and S-ethyl-L-cysteine (10 μmol/kg, IV). The data are shown as mean ± SEM. There were no between-group differences for any study (*p* > 0.05, for all comparisons).

**Supplementary Table S2.** Maximal changes in ventilatory parameters during hypoxic gas challenge

|  |  |  |  |  | **Hypoxic gas (10% O_2_, 90% N_2_) challenge (min)** | | | | |
| --- | --- | --- | --- | --- | --- | --- | --- | --- | --- |
| **Parameter** |  | **Group** | **Pre** |  | **1** | **2.5** | **5** | **7.5** | **10** |
| Freq, breaths/min |  | SHAM | 111 ± 2 |  | 208 ± 8 | 215 ± 6 | 204 ± 6 | 195 ± 8 | 184 ± 7 |
|  |  | CSNX | 103 ± 2 |  | 108 ± 2 | 116 ± 3 | 116 ± 2 | 114 ± 3 | 110 ± 2 |
| TV, ml |  | SHAM | 2.34 ± 0.03 |  | 2.66 ± 0.05 | 3.30 ± 0.08 | 3.67 ± 0.14 | 3.89 ± 0.19 | 3.95 ± 0.22 |
|  |  | CSNX | 2.14 ± 0.05* |  | 2.23 ± 0.06 | 2.33 ± 0.06 | 2.42 ± 0.06 | 2.46 ± 0.05 | 2.43 ± 0.05 |
| MV, ml/min |  | SHAM | 260 ± 5 |  | 552 ± 19 | 710 ± 33 | 726 ± 47 | 764 ± 58 | 731 ± 60 |
|  |  | CSNX | 221 ± 5* |  | 241 ± 7 | 270 ± 10 | 281 ± 6 | 279 ± 6 | 266 ± 5 |
|  |  |  |  |  | **Hypoxic gas (10% O_2_, 90% N_2_) challenge (min)** | | | | |
| **Parameter** |  | **Group** | **Pre** |  | **1** | **2.5** | **5** | **7.5** | **10** |
| Freq, breaths/min |  | VEH | 106 ± 3 |  | 208 ± 12 | 205 ± 11 | 188 ± 8 | 182 ± 9 | 175 ± 8 |
|  |  | CAP | 109 ± 3 |  | 114 ± 3 | 119 ± 3 | 118 ± 3 | 118 ± 3 | 117 ± 3 |
| TV, ml |  | VEH | 2.48 ± 0.08 |  | 2.74 ± 0.08 | 3.27 ± 0.11 | 3.79 ± 0.07 | 3.92 ± 0.08 | 3.95 ± 0.10 |
|  |  | CAP | 2.49 ± 0.08 |  | 2.51 ± 0.09 | 2.64 ± 0.10 | 2.66 ± 0.10 | 2.60 ± 0.09 | 2.59 ± 0.09 |
| MV, ml/min |  | VEH | 264 ± 9 |  | 567 ± 30 | 668 ± 36 | 714 ± 40 | 716 ± 49 | 692 ± 38 |
|  |  | CAP | 270 ± 7 |  | 284 ± 10 | 312 ± 8 | 315 ± 9 | 307 ± 7 | 303 ± 10 |
|  |  |  |  |  | **Hypoxic gas (10% O_2_, 90% N_2_) challenge (min)** | | | | |
| **Parameter** |  | **Group** | **Pre** |  | **1** | **2.5** | **5** | **7.5** | **10** |
| Freq, breaths/min |  | VEH | 110 ± 3 |  | 209 ± 12 | 212 ± 9 | 203± 9 | 192 ± 9 | 178 ± 6 |
|  |  | L-SMC + L-SEC | 103 ± 2 |  | 112 ± 4 | 117 ± 3 | 118 ± 4 | 114 ± 2 | 117 ± 3 |
| TV, ml |  | VEH | 2.31 ± 0.05 |  | 2.60 ± 0.07 | 3.29 ± 0.06 | 3.47 ± 0.11 | 3.66 ± 0.15 | 3.81 ± 0.18 |
|  |  | L-SMC + L-SEC | 2.17 ± 0.05 |  | 2.28 ± 0.06 | 2.31 ± 0.05 | 2.35 ± 0.04 | 2.37 ± 0.05 | 2.34 ± 0.05 |
| MV, ml/min |  | VEH | 254 ± 9 |  | 545 ± 34 | 698 ± 30 | 705 ± 42 | 703 ± 47 | 681 ± 44 |
|  |  | L-SMC + L-SEC | 225 ± 8* |  | 255 ± 9 | 270 ± 7 | 276 ± 9 | 269 ± 7 | 273 ± 8 |

Freq, frequency of breathing; TV, tidal volume; MV, minute ventilation. SHAM, sham-operated rats; CSNX, rats with bilateral carotid sinus nerve transection; VEH, vehicle-treated rats; CAP, capsaicin-treated rats; L-SMC + L-SEC, rats that were receiving co-infusions of S-methyl-L-cysteine (10 μmol/kg, IV) and S-ethyl-L-cysteine (10 μmol/kg, IV). See Supplemental Table 1 for rat numbers per group. The data are shown as mean ± SEM. **p* < 0.05, Pre-values in treatment groups *versus* Pre-values in SHAM/VEH rats.

**Supplementary Table S3.** Maximal changes in ventilatory parameters elicited by L-CSNO and L-GSNO in SHAM or CSNX rats

| **L-CSNO** |  |  | **SHAM rats** | | |  | **CSNX rats** | |
| --- | --- | --- | --- | --- | --- | --- | --- | --- |
| **Parameter** |  | **Dose** | **Pre** |  | **Post** |  | **Pre** | **Post** |
| Freq, breaths/min |  | 2.5 | 114 ± 2 |  | 117 ± 2 |  | 105 ± 2* | 106 ± 2 |
|  |  | 5 | 115 ± 2 |  | 125 ± 2 |  | 104 ± 2 | 106 ± 2 |
|  |  | 10 | 115 ± 2 |  | 130 ± 3 |  | 104 ± 2 | 107 ± 2 |
|  |  | 25 | 114 ± 3 |  | 138 ± 4 |  | 104 ± 2 | 110 ± 2 |
|  |  | 50 | 114 ± 2 |  | 151 ± 4 |  | 104 ± 2 | 113 ± 2 |
| TV, ml |  | 2.5 | 2.44 ± 0.04 |  | 2.45 ± 0.05 |  | 2.21 ± 0.03* | 2.20 ± 0.03 |
|  |  | 5 | 2.45 ± 0.05 |  | 2.62 ± 0.07 |  | 2.21 ± 0.03 | 2.21 ± 0.02 |
|  |  | 10 | 2.42 ± 005 |  | 2.77 ± 0.08 |  | 2.21 ± 0.03 | 2.24 ± 0.04 |
|  |  | 25 | 2.44 ± 0.05 |  | 3.21 ± 0.10 |  | 2.20 ± 0.03 | 2.26 ± 0.05 |
|  |  | 50 | 2.43 ± 0.05 |  | 3.40 ± 0.15 |  | 2.22 ± 0.03 | 2.32 ± 0.06 |
| MV, ml/min |  | 2.5 | 278 ± 5 |  | 295 ± 7 |  | 232 ± 6* | 234 ± 6 |
|  |  | 5 | 283 ± 7 |  | 329 ± 10 |  | 230 ± 6 | 235 ± 6 |
|  |  | 10 | 279 ± 7 |  | 360 ± 12 |  | 231 ± 6 | 240 ± 7 |
|  |  | 25 | 277 ± 8 |  | 443 ± 19 |  | 228 ± 6 | 249 ± 9 |
|  |  | 50 | 278 ± 8 |  | 516 ± 30 |  | 230 ± 6 | 262 ± 10 |
| **L-GSNO** |  |  | **SHAM rats** | | |  | **CSNX rats** | |
| **Parameter** |  | **Dose** | **Pre** |  | **Post** |  | **Pre** | **Post** |
| Freq, breaths/min |  | 5 | 112 ± 2 |  | 117 ± 2 |  | 100 ± 2* | 101 ± 2 |
|  |  | 10 | 112 ± 2 |  | 123 ± 3 |  | 100 ± 2 | 101 ± 2 |
|  |  | 25 | 111 ± 2 |  | 130 ± 3 |  | 101 ± 2 | 104 ± 2 |
|  |  | 50 | 112 ± 2 |  | 138 ± 3 |  | 100 ± 2 | 105 ± 2 |
|  |  | 75 | 113 ± 2 |  | 153 ± 5 |  | 101 ± 2 | 108 ± 3 |
| TV, ml |  | 5 | 2.43 ± 0.05 |  | 2.46 ± 0.06 |  | 2.21 ± 0.03* | 2.22 ± 0.04 |
|  |  | 10 | 2.42 ± 0.05 |  | 2.62 ± 0.05 |  | 2.22 ± 0.04 | 2.23 ± 0.03 |
|  |  | 25 | 2.42 ± 0.05 |  | 2.81 ± 0.06 |  | 2.21 ± 0.03 | 2.27 ± 0.04 |
|  |  | 50 | 2.43 ± 0.05 |  | 3.14 ± 0.08 |  | 2.21 ± 0.04 | 2.35 ± 0.04 |
|  |  | 75 | 2.40 ± 0.05 |  | 3.45 ± 0.09 |  | 2.19 ± 0.04 | 2.36 ± 0.05 |
| MV, ml/min |  | 5 | 272 ± 6 |  | 286 ± 7 |  | 222 ± 5* | 225 ± 7 |
|  |  | 10 | 272 ± 6 |  | 322 ± 8 |  | 221 ± 5 | 226 ± 6 |
|  |  | 25 | 270 ± 5 |  | 367 ± 12 |  | 223 ± 5 | 236 ± 6 |
|  |  | 50 | 271 ± 6 |  | 435 ± 17 |  | 221 ± 6 | 245 ± 6 |
|  |  | 75 | 269 ± 5 |  | 530 ± 24 |  | 221 ± 7 | 256 ± 8 |

All doses are nmol/kg, injected intra-arterially; L-CSNO, S-nitroso-L-cysteine; L-GSNO, S-nitroso-L-glutathione; Freq, frequency of breathing; TV, tidal volume; MV, minute ventilation. SHAM, sham-operated rats; CSNX, bilateral carotid sinus nerve transection. See Supplemental Table 1 for rat numbers per group. The data are shown as mean ± SEM. **p* < 0.05, Pre-values in CSNX rats *versus* Pre-values in SHAM rats.

**Supplementary Table S4.** Maximal changes in ventilatory parameters elicited by L-CSNO and L-GSNO in VEH or CAP rats

| **L-CSNO** |  |  | **VEH rats** | | |  | **CAP rats** | |
| --- | --- | --- | --- | --- | --- | --- | --- | --- |
| **Parameter** |  | **Dose** | **Pre** |  | **Post** |  | **Pre** | **Post** |
| Freq, breaths/min |  | 2.5 | 112 ± 2 |  | 114 ± 2 |  | 112 ± 2 | 112 ± 2 |
|  |  | 5 | 112 ± 2 |  | 122 ± 2 |  | 111 ± 2 | 113 ± 2 |
|  |  | 10 | 113 ± 2 |  | 129 ± 3 |  | 112 ± 3 | 115 ± 2 |
|  |  | 25 | 112 ± 3 |  | 139 ± 3 |  | 113 ± 2 | 116 ± 2 |
|  |  | 50 | 112 ± 2 |  | 152 ± 5 |  | 111 ± 2 | 118 ± 2 |
| TV, ml |  | 2.5 | 2.40 ± 0.04 |  | 2.45 ± 0.05 |  | 2.44 ± 0.05 | 2.45 ± 0.05 |
|  |  | 5 | 2.38 ± 0.05 |  | 2.58 ± 0.07 |  | 2.45 ± 0.05 | 2.45 ± 0.05 |
|  |  | 10 | 2.39 ± 0.05 |  | 2.78 ± 0.07 |  | 2.46 ± 0.06 | 2.49 ± 0.06 |
|  |  | 25 | 2.39 ± 0.05 |  | 3.11 ± 0.07 |  | 2.45 ± 0.05 | 2.53 ± 0.05 |
|  |  | 50 | 2.40 ± 0.05 |  | 3.46 ± 0.13 |  | 2.47 ± 0.05 | 3.58 ± 0.06 |
| MV, ml/min |  | 2.5 | 269 ± 6 |  | 280 ± 8 |  | 272 ± 8 | 275 ± 8 |
|  |  | 5 | 267 ± 7 |  | 314 ± 9 |  | 273 ± 9 | 277 ± 9 |
|  |  | 10 | 268 ± 7 |  | 359 ± 13 |  | 275 ± 9 | 286 ± 8 |
|  |  | 25 | 269 ± 6 |  | 435 ± 18 |  | 275 ± 9 | 293 ± 7 |
|  |  | 50 | 269 ± 8 |  | 527 ± 26 |  | 276 ± 9 | 306 ± 8 |
| **L-GSNO** |  |  | **VEH rats** | | |  | **CAP rats** | |
| **Parameter** |  | **Dose** | **Pre** |  | **Post** |  | **Pre** | **Post** |
| Freq, breaths/min |  | 5 | 112 ± 2 |  | 116 ± 3 |  | 113 ± 2 | 101 ± 2 |
|  |  | 10 | 113 ± 2 |  | 123 ± 2 |  | 113 ± 2 | 114 ± 2 |
|  |  | 25 | 113 ± 3 |  | 129 ± 4 |  | 114 ± 2 | 115 ± 2 |
|  |  | 50 | 112 ± 2 |  | 139 ± 5 |  | 113 ± 2 | 119 ± 3 |
|  |  | 75 | 114 ± 2 |  | 155 ± 5 |  | 114 ± 2 | 123 ± 3 |
| TV, ml |  | 5 | 2.42 ± 0.04 |  | 2.44 ± 0.04 |  | 2.45 ± 0.06 | 2.46 ± 0.05 |
|  |  | 10 | 2.42 ± 0.04 |  | 2.62 ± 0.04 |  | 2.43 ± 0.06 | 2.47 ± 0.07 |
|  |  | 25 | 2.44 ± 0.03 |  | 2.85 ± 0.06 |  | 2.46 ± 0.06 | 2.51 ± 0.06 |
|  |  | 50 | 2.44 ± 0.04 |  | 3.13 ± 0.08 |  | 2.45 ± 0.05 | 2.55 ± 0.06 |
|  |  | 75 | 2.42 ± 0.04 |  | 3.48 ± 0.11 |  | 2.45 ± 0.06 | 2.56 ± 0.05 |
| MV, ml/min |  | 5 | 271 ± 6 |  | 284 ± 6 |  | 276 ± 8 | 281 ± 7 |
|  |  | 10 | 273 ± 6 |  | 322 ± 7 |  | 275 ± 8 | 283 ± 10 |
|  |  | 25 | 275 ± 6 |  | 369 ± 15 |  | 277 ± 8 | 288 ± 9 |
|  |  | 50 | 274 ± 7 |  | 436 ± 20 |  | 278 ± 8 | 304 ± 12 |
|  |  | 75 | 274± 6 |  | 542 ± 28 |  | 278 ± 8 | 314 ± 9 |

All doses are nmol/kg, injected intra-arterially; L-CSNO, S-nitroso-L-cysteine; L-GSNO, S-nitroso-L-glutathione; Freq, frequency of breathing; TV, tidal volume; MV, minute ventilation. VEH, vehicle-treated rats; CAP, capsaicin-treated rats. See Supplemental Table 1 for rat numbers per group. The data are shown as mean ± SEM. **p* < 0.05, Pre-values in CAP rats *versus* Pre-values in VEH rats.

**Supplementary Table S5.** Maximal changes in ventilatory parameters elicited by L-CSNO and L-GSNO in VEH or L-SMC + L-SEC rats

| **L-CSNO** |  |  | **VEH rats** | | |  | **L-SMC + L-SEC rats** | |
| --- | --- | --- | --- | --- | --- | --- | --- | --- |
| **Parameter** |  | **Dose** | **Pre** |  | **Post** |  | **Pre** | **Post** |
| Freq, breaths/min |  | 2.5 | 111 ± 2 |  | 113 ± 3 |  | 105 ± 2 | 106 ± 2 |
|  |  | 5 | 111 ± 3 |  | 121 ± 3 |  | 104 ± 2 | 107 ± 2 |
|  |  | 10 | 112 ± 2 |  | 128 ± 4 |  | 105 ± 3 | 108 ± 2 |
|  |  | 25 | 111 ± 2 |  | 135 ± 5 |  | 105 ± 2 | 111 ± 2 |
|  |  | 50 | 112 ± 2 |  | 147 ± 5 |  | 103 ± 2 | 113 ± 2 |
| TV, ml |  | 2.5 | 2.38 ± 0.06 |  | 2.39 ± 0.05 |  | 2.26 ± 0.04 | 2.26 ± 0.04 |
|  |  | 5 | 2.39 ± 0.06 |  | 2.57 ± 0.06 |  | 2.26 ± 0.04 | 2.27 ± 0.03 |
|  |  | 10 | 2.38 ± 0.06 |  | 2.70 ± 0.09 |  | 2.25 ± 0.04 | 2.28 ± 0.04 |
|  |  | 25 | 2.38 ± 0.07 |  | 3.06 ± 0.11 |  | 2.23 ± 0.04 | 2.33 ± 0.05 |
|  |  | 50 | 2.42 ± 0.06 |  | 3.33 ± 0.16 |  | 2.22 ± 0.04 | 2.36 ± 0.04 |
| MV, ml/min |  | 2.5 | 265 ± 10 |  | 269 ± 9 |  | 237 ± 6* | 240 ± 7 |
|  |  | 5 | 266 ± 10 |  | 311 ± 12 |  | 235 ± 6 | 242 ± 5 |
|  |  | 10 | 267 ± 10 |  | 346 ± 21 |  | 236 ± 6 | 247 ± 7 |
|  |  | 25 | 265 ± 10 |  | 412 ± 17 |  | 234 ± 6 | 257 ± 7 |
|  |  | 50 | 270 ± 10 |  | 492 ± 36 |  | 230 ± 5 | 267 ± 8 |
| **L-GSNO** |  |  | **VEH rats** | | |  | **L-SMC + L-SEC rats** | |
| **Parameter** |  | **Dose** | **Pre** |  | **Post** |  | **Pre** | **Post** |
| Freq, breaths/min |  | 5 | 113 ± 2 |  | 117 ± 2 |  | 105 ± 2* | 108 ± 2 |
|  |  | 10 | 113 ± 2 |  | 122 ± 2 |  | 104 ± 2 | 114 ± 2 |
|  |  | 25 | 112 ± 3 |  | 130 ± 2 |  | 104 ± 2 | 121 ± 3 |
|  |  | 50 | 114 ± 2 |  | 139 ± 3 |  | 104 ± 2 | 135 ± 5 |
|  |  | 75 | 113 ± 2 |  | 153 ± 4 |  | 103 ± 2 | 145 ± 5 |
| TV, ml |  | 5 | 2.44 ± 0.04 |  | 2.46 ± 0.04 |  | 2.28 ± 0.05* | 2.30 ± 0.05 |
|  |  | 10 | 2.43 ± 0.04 |  | 2.63 ± 0.03 |  | 2.27 ± 0.05 | 2.45 ± 0.06 |
|  |  | 25 | 2.44 ± 0.04 |  | 2.82 ± 0.06 |  | 2.27 ± 0.04 | 2.65 ± 0.07 |
|  |  | 50 | 2.43 ± 0.03 |  | 3.10 ± 0.06 |  | 2.28 ± 0.04 | 2.92 ± 0.10 |
|  |  | 75 | 2.42 ± 0.04 |  | 3.44 ± 0.09 |  | 2.27 ± 0.03 | 3.26 ± 0.11 |
| MV, ml/min |  | 5 | 275 ± 5 |  | 289 ± 6 |  | 239 ± 9* | 248 ± 10 |
|  |  | 10 | 274 ± 5 |  | 322 ± 5 |  | 237 ± 9 | 280 ± 11 |
|  |  | 25 | 275 ± 5 |  | 366 ± 10 |  | 237 ± 8 | 321 ± 14 |
|  |  | 50 | 275 ± 7 |  | 431 ± 15 |  | 236 ± 9 | 393 ± 16 |
|  |  | 75 | 272 ± 5 |  | 527 ± 22 |  | 233 ± 7 | 475 ± 27 |

All doses are nmol/kg, injected intra-arterially; L-CSNO, S-nitroso-L-cysteine; L-GSNO, S-nitroso-L-glutathione; Freq, frequency of breathing; TV, tidal volume; MV, minute ventilation. VEH, vehicle-treated rats; L-SMC + L-SEC, rats that were receiving co-infusions of S-methyl-L-cysteine (10 μmol/kg, IV) and S-ethyl-L-cysteine (10 μmol/kg, IV). See Supplemental Table 1 for rat numbers per group. The data are shown as mean ± SEM. **p* < 0.05, Pre-values in L-SMC + L-SEC rats *versus* Pre-values in VEH rats.

**Supplementary Table S6.** Changes in ventilatory parameters elicited by infusion of L-SMC + L-SEC

| **Parameter** |  | **Treatment** |  | **Pre** |  | **Post L-SMC+L-SEC** |  | **%Change** |
| --- | --- | --- | --- | --- | --- | --- | --- | --- |
| **Hypoxia Study** | | | | | | | | |
| Freq, breaths/min |  | VEH |  | 109.4 ± 2.5 |  | 109.7 ± 2.5 |  | +0.2 ± 0.3 |
|  |  | L-SMC + L-SEC |  | 111.6 ± 2.4 |  | 103.2 ± 2.2 |  | -7.5 ± 1.6* |
| TV, ml |  | VEH |  | 2.31 ± 0.05 |  | 2.31 ± 0.06 |  | +0.1 ± 0.5 |
|  |  | L-SMC + L-SEC |  | 2.33 ± 0.04 |  | 2.17 ± 0.05 |  | -6.6 ± 0.8* |
| MV, ml/min |  | VEH |  | 253.2 ± 8.7 |  | 253.9 ± 8.9 |  | +0.2 ± 0.4 |
|  |  | L-SMC + L-SEC |  | 259.4 ± 6.8 |  | 224.5 ± 7.6 |  | -13.6 ± 1.8* |
| **L-CSNO Study** | | | | | | | | |
| Freq, breaths/min |  | VEH |  | 111.6 ± 2.6 |  | 111.0 ± 2.4 |  | 0.5 ± 0.6 |
|  |  | L-SMC + L-SEC |  | 110.3 ± 1.8 |  | 104.9 ± 1.8 |  | -4.9 ± 0.5* |
| TV, ml |  | VEH |  | 2.40 ± 0.06 |  | 2.38 ± 0.06 |  | -0.8 ± 0.5 |
|  |  | L-SMC + L-SEC |  | 2.41 ± 0.05 |  | 2.26 ± 0.04 |  | -5.9 ± 0.7* |
| MV, ml/min |  | VEH |  | 268.3 ± 10.1 |  | 264.9 ± 9.9 |  | -2.3 ± 1.9 |
|  |  | L-SMC + L-SEC |  | 264.9 ± 7.2 |  | 236.9 ± 6.0 |  | -10.5 ± 1.6* |
| **L-GSNO Study** | | | | | | | | |
| Freq, breaths/min |  | VEH |  | 113.1 ± 1.8 |  | 112.9 ± 2.2 |  | -0.2 ± 0.8 |
|  |  | L-SMC + L-SEC |  | 110.3 ± 2.3 |  | 104.6 ± 2.3 |  | -5.8 ± 0.6* |
| TV, ml |  | VEH |  | 2.43 ± 0.06 |  | 2.44 ± 0.04 |  | +0.7 ± 0.9 |
|  |  | L-SMC + L-SEC |  | 2.42 ± 0.05 |  | 2.28 ± 0.05 |  | -5.9 ± 0.8* |
| MV, ml/min |  | VEH |  | 274.5 ± 7.0 |  | 275.4 ± 5.3 |  | +0.5 ± 1.3 |
|  |  | L-SMC + L-SEC |  | 267.8 ± 10.8 |  | 238.9 ± 8.9 |  | -10.7 ± 1.8* |

L-SMC, S-methyl-L-cysteine (10 μmol/kg/min); L-SEC, S-ethyl-L-cysteine (10 μmol/kg/min); Freq, frequency of breathing; TV, tidal volume; MV, minute ventilation. VEH, vehicle-treated rats. See Supplemental Table 1 for rat numbers per group. The data are shown as mean ± SEM. **p* < 0.05, L-SMC + L-SEC *versus* Pre-values.

**Supplementary Figure S1**

**A.**

**D.**

**B.**

**E.**

**F.**

**C.**

**Supplementary Figure S1.** Arithmetic changes in frequency of breathing (**left panels**) and tidal volume (**right panels**) during a hypoxic gas challenge (10% O_2_, 90% N_2_) for 10 min. **Panels A and D:** Adult sham-operated (SHAM) rats (n = 6) and those with bilateral carotid sinus nerve transection (CSNX) (n = 6). **Panels B and E:** Adult rats treated as neonates with vehicle (VEH; n = 9) or capsaicin (CAP; 50 mg/kg, SC; n = 9). **Panel C and F:** Adult rats receiving a continuous intravenous infusion of vehicle (VEH; 20 μL/min, IV; n = 9) or S-methyl-L-cysteine (L-SMC; 10 μmol/min, IV; n = 9) plus S-ethyl-L-cysteine (L-SEC; 10 μmol/min, IV; n = 9). The data are shown as mean ± SEM. **p* < 0.05, significant response. †*p* < 0.05, CSNX *versus* SHAM, CAP *versus* VEH, L-SMC + L-SEC *versus* VEH.

**Supplementary Figure S2**

**F.**

**D.**

**E.**

**B.**

**C.**

**A.**

**Supplementary Figure S2.** Arithmetic changes in frequency of breathing (**left panels**) and tidal volume (**right panels**) elicited by intra-arterial (IA) injections of S-nitroso-L-cysteine (L-CSNO). **Panels A and D:** Adult sham-operated (SHAM) rats (n = 6) and those with bilateral carotid sinus nerve transection (CSNX) (n = 6). **Panels B and E:** Adult rats treated as neonates with vehicle (VEH; n = 9) or capsaicin (CAP; 50 mg/kg, SC; n = 9). **Panel C and F:** Adult rats receiving a continuous intravenous infusion of vehicle (VEH; 20 μL/min, IV; n = 9) or S-methyl-L-cysteine (L-SMC; 10 μmol/min, IV; n = 9) plus S-ethyl-L-cysteine (L-SEC; 10 μmol/min, IV; n = 9). The data are shown as mean ± SEM. **p* < 0.05, significant response. †*p* < 0.05, CSNX *versus* SHAM, CAP *versus* VEH, L-SMC + L-SEC *versus* VEH.

**Supplementary Figure S3**

**D.**

**A.**

**E.**

**B.**

**F.**

**C.**

**Supplementary Figure S3.** Arithmetic changes in frequency of breathing (**left panels**) and tidal volume (**right panels**) elicited by intra-arterial (IA) injections of S-nitroso-L-glutathione (L-GSNO). **Panels A and D:** Adult sham-operated (SHAM) rats (n = 6) and those with bilateral carotid sinus nerve transection (CSNX) (n = 6). **Panels B and E:** Adult rats treated as neonates with vehicle (VEH; n = 9) or capsaicin (CAP; 50 mg/kg, SC; n = 9). **Panel C and F:** Adult rats receiving a continuous intravenous infusion of vehicle (VEH; 20 μL/min, IV; n = 9) or S-methyl-L-cysteine (L-SMC; 10 μmol/min, IV; n = 9) plus S-ethyl-L-cysteine (L-SEC; 10 μmol/min, IV; n = 9). The data are shown as mean ± SEM. **p* < 0.05, significant response. †*p* < 0.05, CSNX *versus* SHAM, CAP *versus* VEH, L-SMC + L-SEC *versus* VEH.
